# Impact of Double Covalent Binding of BV in NIR FPs on Their Spectral and Physicochemical Properties

**DOI:** 10.3390/ijms23137347

**Published:** 2022-07-01

**Authors:** Olga V. Stepanenko, Irina M. Kuznetsova, Konstantin K. Turoverov, Olesya V. Stepanenko

**Affiliations:** Laboratory of Structural Dynamics, Stability and Folding of Proteins, Institute of Cytology, Russian Academy of Sciences, 4 Tikhoretsky ave., 194064 St. Petersburg, Russia; sov@incras.ru (O.V.S.); imk@incras.ru (I.M.K.); lvs@incras.ru (O.V.S.)

**Keywords:** bacterial phytochrome, near-infrared biomarkers, stability, proteolytic degradation, fluorescence quantum yield, biliverdin

## Abstract

Understanding the photophysical properties and stability of near-infrared fluorescent proteins (NIR FPs) based on bacterial phytochromes is of great importance for the design of efficient fluorescent probes for use in cells and in vivo. Previously, the natural ligand of NIR FPs biliverdin (BV) has been revealed to be capable of covalent binding to the inherent cysteine residue in the PAS domain (Cys15), and to the cysteine residue introduced into the GAF domain (Cys256), as well as simultaneously with these two residues. Here, based on the spectroscopic analysis of several NIR FPs with both cysteine residues in PAS and GAF domains, we show that the covalent binding of BV simultaneously with two domains is the reason for the higher quantum yield of BV fluorescence in these proteins as a result of rigid fixation of the chromophore in their chromophore-binding pocket. We demonstrate that since the attachment sites are located in different regions of the polypeptide chain forming a figure-of-eight knot, their binding to BV leads to shielding of many sites of proteolytic degradation due to additional stabilization of the entire protein structure. This makes NIR FPs with both cysteine residues in PAS and GAF domains less susceptible to cleavage by intracellular proteases.

## 1. Introduction

Genetically encoded protein fluorescent markers are routinely used to study biological processes by selectively labeling cellular targets (proteins, organelles) involved in these processes [1,2]. An indisputable advantage of near-infrared (NIR) biomarkers is the possibility of their use in experiments on living model animals since the absorption and fluorescence spectra of these proteins fall within the “transparency window” of biological tissues—the spectral region in which cell components no longer absorb and water does not yet absorb [3].

Most of the currently available NIR biomarkers are developed from NIR fluorescent proteins (NIR FPs), which consist of chromophore-binding domains composed of two full-length bacterial phytochrome domains, PAS (Per-ARNT-Sim) and GAF (cGMP PDE/AC/FhlA) [1,4]. The choice of bacterial phytochromes for the development of NIR FPs is largely due to the fact that the natural ligand of bacterial phytochromes is biliverdin (BV), a product of endogenous synthesis from heme, which is always present in cells [5].

When BV interacts with NIR FP, it incorporates into the so-called chromophore-binding pocket formed by the PAS and GAF domains and covalently binds to inherent Cys15 in the N-terminal region of the PAS domain (Cys^PAS^). However, these NIR FPs have a very low fluorescence quantum yield. To increase the fluorescence quantum yield, a mutant form of NIR FP was created that has a cysteine residue in the GAF domain (Cys^GAF^), in a position equivalent to the attachment site of the plant and cyanobacteria photoreceptor (Cys256). The spectral properties of dimeric NIR FPs with all possible combinations of Cys^PAS^ and Cys^GAF^ have been studied [6,7]. It has been shown that the highest fluorescence quantum yield among BV-bearing NIR FPs exhibits those of them containing both cysteine residues, Cys^PAS^ and Cys^GAF^. Crystallography of the monomeric protein miRFP670, containing both Cys^PAS^ and Cys^GAF^, has revealed the covalent attachment of BV to these two residues simultaneously [8]. This type of BV attachment is believed to be relevant for the highest fluorescence quantum yield of BV-containing NIR FPs with Cys^PAS^ and Cys^GAF^ compared to other BV-containing NIR FPs.

To date, this assumption has been confirmed only by analyzing the Raman spectra of the iRFP682 (by the band shift at 1656 cm^−1^) [9]. We were surprised that the covalent binding of BV to two cysteine residues of NIR FPs was not confirmed directly by mass spectroscopy. In this regard, we decided to try to obtain the samples of several NIR FPs, in which BV is covalently bound simultaneously to Cys^PAS^ and Cys^GAF^, and to more accurately determine the spectral characteristics of these proteins. These NIR FPs include a monomeric protein BphP1-FP, and dimeric proteins iRFP713/V256C and iRFP670 derived from bacterial phytochromes *Rp*BphP1, *Rp*BphP2, and *Rp*BphP6 from *Rhodopseudomonas palustris*, respectively. In this work, we analyzed the resistance of NIR FPs containing two cysteine residues to proteases and revealed the reasons for their high resistance to proteolytic cleavage.

## 2. Results and Discussion

### 2.1. Preparation of NIR FPs Enriched in Cys^GAF^-BV-Cys^PAS^

**Figure 1:**
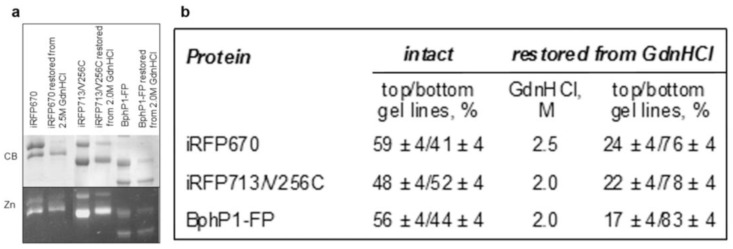
The content of two types of molecules containing BV-Cys^GAF^ or Cys^GAF^-BV-Cys^PAS^ in NIR FPs with two cysteine residues in the PAS and GAF domains in their original state and treated with GdnHCl at moderate concentrations followed by restoration. (**a**) The SDS PAGE of NIR FP samples followed by staining with Coomassie blue (CB) and detection of zinc-induced fluorescence (Zn). (**b**) The proportion of NIR FP monomers that contain BV-Cys^GAF^ or Cys^GAF^-BV-Cys^PAS^.

Monomeric [8] and dimeric (Figure 1) NIR FPs with two cysteine residues in the PAS and GAF domains can bind their chromophore, BV, in two different ways: covalently through the C3^2^ atom of BV to Cys^GAF^ (BV-Cys^GAF^) and covalently simultaneously through the C3^1^ atom of BV to Cys^GAF^ and the C3^2^ atom of BV to Cys^PAS^ (Cys^GAF^-BV-Cys^PAS^). The ratio of NIR FP molecules, in which the mode of BV binging differs, is approximately equimolar. The presence of two types of protein molecules in approximately equal amounts is evidenced by two bands of the same total intensity on the gels visualizing the separation of these proteins under denaturing conditions (Figure 1). At the same time, the band of higher electrophoretic mobility corresponds to NIR FP monomers containing BV covalently bound to two cysteine residues simultaneously. The covalent attachment of BV simultaneously to the two cysteine residues in the PAS and GAF domains of the NIR FP is supposed to result in an inability to untie the unique figure-eight knot [10] encompassing these domains [8], which makes the denatured state of the NIR FP rather compact. 

To analyze the effect of covalent BV binding simultaneously with two cysteine residues on the spectral properties of NIR FPs, we obtained protein samples with increased content of Cys^GAF^-BV-Cys^PAS^. This was achieved using the procedure proposed by Buhrke for iRFP682 [9], which was modified by taking into account the results of our studies of the unfolding–refolding processes and the structural stability of NIR FPs [7,11]. We have previously shown that dimeric and monomeric NIR FPs containing both cysteine residues in the PAS and GAF domains are more resistant to chemical denaturing agents compared to those NIR FPs with cysteine residues capable of covalent binding the chromophore either in the PAS domain or in the GAF domain or missing [7,11]. The stabilization of NIR FPs in the presence of two cysteine residues is likely attributed to the binding of two regions of the polypeptide chain at the formation of Cys^GAF^-BV-Cys^PAS^, which fastens both domains of the proteins together [12]. With this in mind, we chose the conditions (denaturing agent concentration) for the incubation of NIR FPs with two cysteine residues under which their monomers containing BV-Cys^GAF^ would predominantly denature, while monomers containing Cys^GAF^-BV-Cys^PAS^ would retain its intact structure. We propose another rather simple approach for the subsequent separation of denatured and native NIR FP molecules instead of the gel filtration [9], based on the fact that the covalent attachment of a chromophore leads to irreversible denaturation of NIR FPs [13]. According to this procedure, after incubation of the analyzed NIR FPs in the presence of 2–2.5 M GdnHCl (Figure 1b), the denaturant is removed from the solution by the method of equilibrium microdialysis. The change in the composition of the buffer stimulates the aggregation of denatured NIR FP molecules due to the accumulation of misfolded forms of the protein. Indeed, the samples of NIR FPs analyzed in this work treated in this way showed the presence of a clearly visible precipitate. This fraction of aggregates in the samples of NIR FPs is easily removed by centrifugation.

After the above-described processing of NIR FPs and their separation by SDS PAGE, the assessment of the total intensity of the bands corresponding to molecules containing BV-Cys^GAF^ and Cys^GAF^-BV-Cys^PAS^ indicated an increase in the content of the latter molecules from 50 to 80% (Figure 1). Thus, according to the procedure described above, NIR FP samples were obtained, the monomers of which contained mainly Cys^GAF^-BV-Cys^PAS^ (Figure 1).

### 2.2. Spectral Properties of NIR FPs Containing Predominantly Cys^GAF^-BV-Cys^PAS^

**Figure 2:**
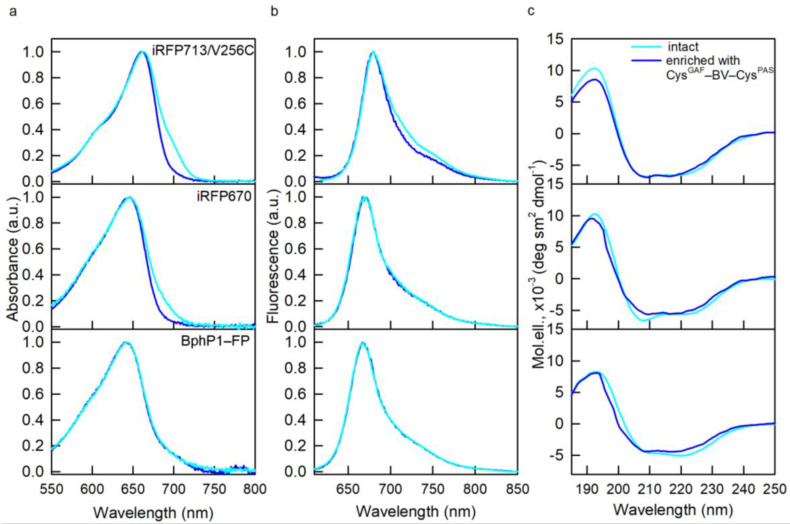
Spectral properties of NIR FPs enriched with Cys^GAF^-BV-Cys^PAS^ (curves in blue). (**a**) Absorption spectra of the chromophore. (**b**) chromophore fluorescence spectra (λ_ex_ = 590 nm). (**c**) CD spectra in the spectral far-UV region. The spectral characteristics of the original NIR FPs containing both cysteine residues in the PAS and GAF domains are also shown (cyan curves).

The spectral properties of NIR FPs enriched in Cys^GAF^-BV-Cys^PAS^ were characterized by absorption, fluorescence spectroscopy, and circular dichroism (CD). The absorption and fluorescence spectra of NIR FPs enriched in Cys^GAF^-BV-Cys^PAS^ almost coincided with the spectra of the original NIR FPs (Figure 2). However, the absorption spectra of dimeric NIR FPs containing predominantly Cys^GAF^-BV-Cys^PAS^ were narrower compared to the spectra of the original proteins. This confirms the data obtained earlier by X-ray diffraction analysis on the structure of BV-Cys^GAF^ and Cys^GAF^-BV-Cys^PAS^ and the identity of a system of conjugated π-bonds in them, which determines the spectral similarity of differently bound chromophore [8].

According to the data of far-UV CD, NIR FPs containing predominantly Cys^GAF^-BV-Cys^PAS^ retain the secondary structure inherent in the original NIR FPs (Figure 2). The tryptophan fluorescence spectra of NIR FPs containing predominantly Cys^GAF^-BV-Cys^PAS^ were blue-shifted by 1–3 nm relative to the spectra of the original proteins (Table 1). The blue shift of the tryptophan spectrum indicates an increase in the rigidity of the microenvironment of protein tryptophan residues and a decrease in their accessibility to the solvent [14,15]. These data testify to some compaction of the structure of NIR FPs containing predominantly Cys^GAF^-BV-Cys^PAS^; however, there was observed no significant change in the spatial structure of these proteins compared to NIR FPs with equimolar content of Cys^GAF^-BV-Cys^PAS^ and BV-Cys^GAF^.

**Figure 3:**
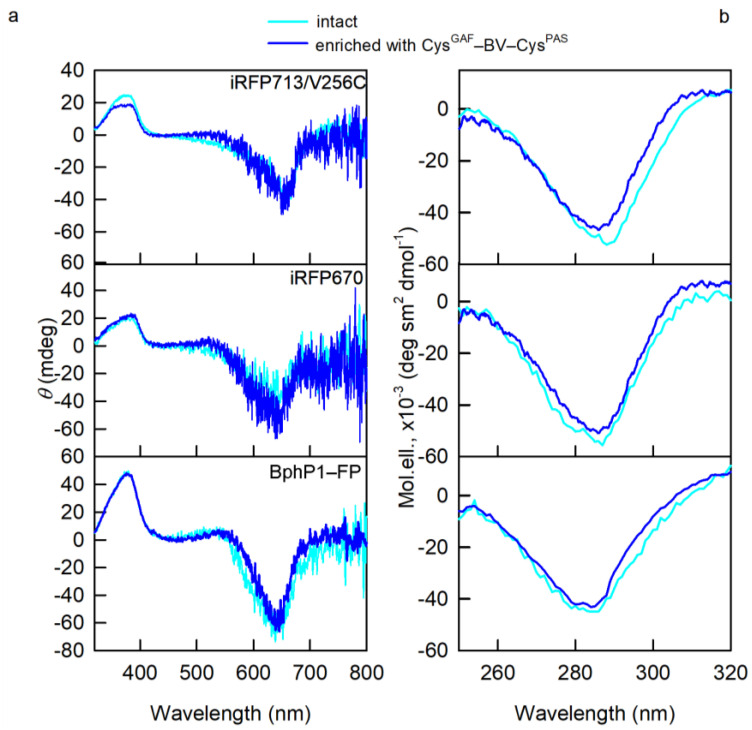
Properties of BV environment in NIR FPs enriched with Cys^GAF^-BV-Cys^PAS^ (curves in blue). (**a**) CD spectra in the visible spectral region (**b**) CD spectra in the near-UV spectral region. The spectral characteristics of the original NIR FPs containing both cysteine residues in the PAS and GAF domains are also shown (cyan curves).

The overlap of the CD spectra in the visible region for NIR FPs containing predominantly Cys^GAF^-BV-Cys^PAS^ (Figure 3) and their initial forms points at the same 15Z-configuration of BV-Cys^GAF^ and Cys^GAF^-BV-Cys^PAS^, which is characteristic of the Pr-form of phytochromes [16,17,18]. Nevertheless, narrower CD spectra in the near UV region of NIR FPs containing predominantly Cys^GAF^-BV-Cys^PAS^ compared to the spectra of the original proteins stand out (Figure 3). This implies a greater rigidity of the microenvironment of Cys^GAF^-BV-Cys^PAS^ compared to BV-Cys^GAF^, since the optical activity of NIR probes in this spectral range is linked to the interaction of the pyrrole rings of their chromophore with aromatic residues of the protein [13,17].

The fluorescence quantum yield of NIR FPs containing predominantly Cys^GAF^-BV-Cys^PAS^ exceeded by 1–2% this characteristic for NIR FPs containing both Cys^GAF^-BV-Cys^PAS^ and BV-Cys^GAF^ in an equimolar ratio (Table 1). An increase in the fluorescence quantum yield of a NIR FP upon its saturation with Cys^GAF^-BV-Cys^PAS^ also correlated with a higher fluorescence lifetime of the protein (Table 1) [19,20].

Taken together, the obtained data confirm that Cys^GAF^-BV-Cys^PAS^ exhibits a higher fluorescence quantum yield compared to BV bound in NIR FPs differently. Our findings demonstrate also that the increased quantum yield of the former is associated with its rigid fixation in the GAF domain of the analyzed NIR FPs, which leads to a decrease in the efficiency of nonradiative deactivation of the chromophore excited state [20,21]. These data are consistent with the results of a study of the structural properties of the iRFP682 chromophore involved in covalent binding to both cysteine residues in the GAF and PAS domains of the protein using resonance Raman spectroscopy [9]. According to data of SDS PAGE (Figure 1), the content of two types of molecules with BV-Cys^GAF^ or Cys^GAF^-BV-Cys^PAS^ may differ slightly for iRFP713/V256C, iRFP670, and BphP1-FP. This could be one of the reasons for the higher quantum yield of iRFP713/V256C compared to BphP1-FP and iRFP670. According to the results of our previous studies, the way BV binds to NIR FPs may depend on the amino acid composition of the GAF domain pocket [22]. The influence of the microenvironment of the BV chromophore in NIR FPs on the dynamics of the chromophore and, consequently, the efficiency of nonradiative deactivation of its excited state should not be ruled out [20,23]. In particular, it is known that the residue at position 204 (numbering of amino acids is given according to the *Rp*BphP2 sequence) is directly involved in the hydrogen bond network with the chromophore and other amino acids in NIR FPs and, in addition, can affect the number of water molecules inside the pocket of the GAF domain, also impacting the proton wire proteins [21,24,25,26,27].

### 2.3. Effect of Cys^GAF^-BV-Cys^PAS^ on NIR FPs Stability in the Cell

Obtaining NIR FPs with increased content of Cys^GAF^-BV-Cys^PAS^ by selective denaturation of monomers with Cys^GAF^-BV in the parent NIR FPs implies that the cross-linking of the PAS and GAF domains through simultaneous binding of cysteine residues in them to the chromophore indeed stabilizes the NIR FP structure. The stability of the biomarker in the cell, in addition to the stability of its structure, is affected by its resistance to the action of proteolytic enzymes. Therefore, we analyzed the influence of proteases, trypsin, and chymotrypsin, on the structure of NIR FPs containing both cysteine residues in the PAS and GAF domains using iRFP670 as an example. Protease-treated samples were analyzed using the Tricine-SDS polyacrylamide gel electrophoresis, used to separate peptides over a wide range of molecular weights [28]. The iRFP670 protein has 33 and 22 cleavage sites of trypsin and chymotrypsin, respectively (Figure S1). The peptide size of iRFP670 processed with proteases is calculated considering the presence of BV differently bound to protein monomers, Cys^GAF^-BV and Cys^GAF^-BV-Cys^PAS^ (Table S1).

We found that only bands corresponding to the target uncleaved protein were visualized on gels of iRFP670 in the holoform after its treatment with proteases (Figure S2, gel 1). The pre-incubation of the protein under denaturing conditions (5 M urea) to destabilize its structure [29,30] did not promote protein cleavage by proteases (Figure S2, gel 1). This indicates a high resistance of iRFP670 in the holoform to the action of proteases.

**Figure 4:**
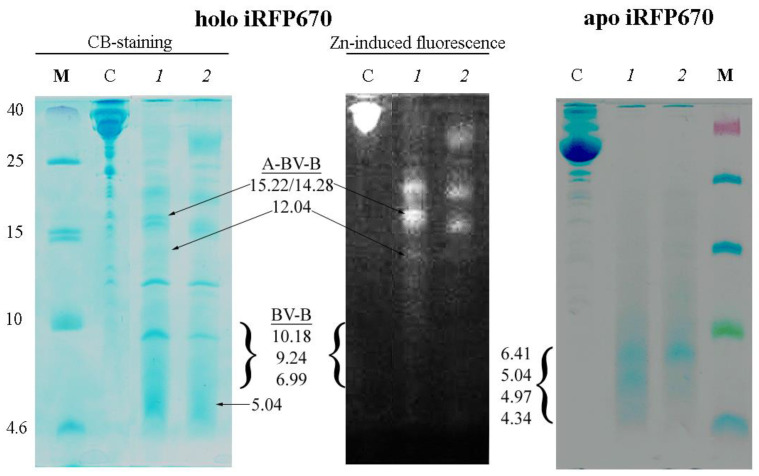
Cleavage of iRFP670 in the holoform (**left panels**) and the apoform (**right panel**) with chymotrypsin. Tricine-SDS PAGE of protein samples followed by staining with Coomassie blue (CB) and detection of zinc-induced fluorescence (Zn). The following samples were loaded into the wells: M—marker peptides, C—control samples of the original protein, 1 and 2—iRFP670 samples cleaved at 4 and 20 °C. The expected size of the detected peptides and the nature of BV binding to them are indicated (see also Table 2).

Partial cleavage of iRFP670 by chymotrypsin, but not trypsin, in the presence of ProteaseMAX™ Surfactant (Trypsin Enhancer) was observed [31,32,33] (Figure S2, gel 2 and 3). It is believed that this chemical increases the efficiency of several proteolytic enzymes by destabilizing the structure of the analyzed proteins and solubilizing them [31]. We found the temperatures of iRFP670 incubation in the presence of chymotrypsin should be lowered to avoid autolysis of the protease (Figure S2, gel 4). In the solution of iRFP670 cleaved with chymotrypsin, peptides were found mainly with a molecular weight of about 14–15 kDa and higher (Figure 4). These peptides are modified with a covalently attached chromophore as evidenced by Zn-induced fluorescence of the corresponding electrophoretic bands (Figure 4). The analyzed solution contained trace amounts of peptides with a molecular weight of about 12 kDa and 7–10 kDa with a covalently attached chromophore in their composition (Figure 4).

**Figure 5:**
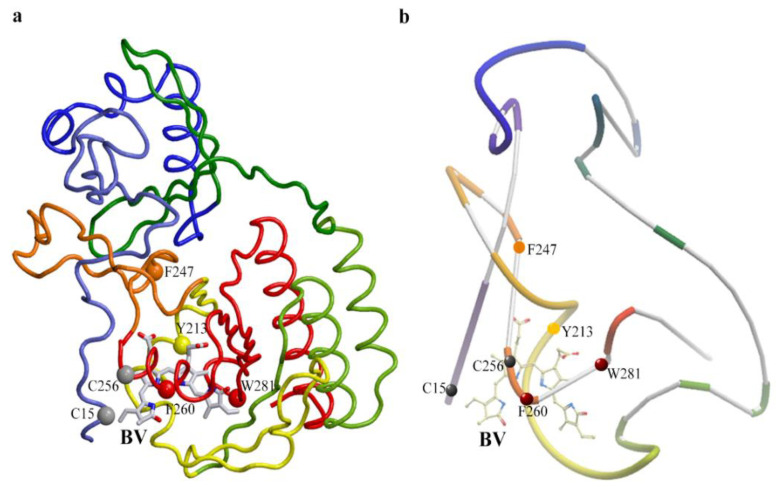
Location of proteolytic cleavage sites in the iRFP670 structure that is potentially inaccessible to chymotrypsin. (**a**) Spatial structure of the protein. (**b**) The schematic representation of the structure of the protein shows the knot topology. The figure was created based on X-ray data of PAS-GAF domains of *Rp*BphP2 protein from *Rhodopseudomonas palustris* (PDB: 4e04 file [34]) using the graphic software VMD (Visual Molecular Dynamics) [35], Raster3D [36] and ICM Pro [37]. Chymotrypsin cleavage sites Y213, F247, F260, and W281 are shown as van der Waals spheres (CA atoms of residues are shown). BV chromophore is displayed by sticks.

To clarify the reasons for partial proteolysis of iRFP670, we analyzed the location of chymotrypsin cleavage sites in the protein using crystallographic data on the structure of phytochromes (Table 2, Figure 5). We discovered potential steric inaccessibility for the action of chymotrypsin at some cleavage sites in the protein sequence. Thus, the inaccessibility of the Y213 cleavage site is likely associated with its location near one of the intersections of the protein polypeptide chain that forms the knot (Figure 5). The F247 cleavage site is located near another intersection of the polypeptide chain in a loop through which the N-terminal segment of the protein is pulled (Figure 5). These two cleavage sites are located N-terminally from the Cys^GAF^ residue. According to the calculation, the solution of iRFP670 not hydrolyzed by chymotrypsin at Y213 and F247 sites should contain peptides with a covalently attached chromophore with a molecular weight of 6.99 (peptide B-BV) and 12.04 kDa (peptide B-BV-peptide A) (Table 2). The protease access to the F260 cleavage site located at the C-terminus of Cys^GAF^ may be limited by the covalent binding of BV in the protein (Figure 5), which will lead to the appearance in the analyzed solution of peptides with a covalently attached chromophore of a size of 9.24 (peptide B-BV) and 14.28 kDa (peptide B-BV-peptide A) (Table 2). The W281 cleavage site which is C-terminal to Cys^GAF^ is buried deep within the protein globule in its GAF domain (Figure 5). In the case of non-hydrolysis of the iRFP670 polypeptide chain at all of the sites listed, including Y213, F247, F260, and W281, the size of the anticipated protein fragments will be 10.18 kDa (peptide B-BV) and 15.22 kDa (peptide B-BV-peptide A) (Table 2). At the cleavage of iRFP670 monomers containing Cys^GAF^-BV, a peptide with a Cys^PAS^ residue without a covalently attached chromophore and with a molecular weight of about 5.04 kDa is also expected (Table S1).

Considering the structural analysis performed, the presence on the gel of intense Zn-positive bands corresponding to peptides of 14–15 kDa, barely pronounced Zn-positive bands corresponding to peptides of 12 kDa and 7–10 kDa, as well as Zn-negative band corresponding to the peptide of 5 kDa can be interpreted in favor of only a marginal cleavage of the protein monomer with Cys^GAF^-BV-Cys^PAS^, and almost complete cleavage of the monomer with Cys^GAF^-BV (Figure 4). Our data are in good agreement with the literature on the presence of two types of chromophore binding in dimeric and monomeric NIR FPs with two cysteine residues Cys^PAS^ and Cys^GAF^ [8,12]. The large size of chromophore-bearing peptides obtained by cleavage of iRFP670 with chymotrypsin also is in good agreement with the assumption of simultaneous covalent attachment of BV to cysteine residues in both domains in NIR FPs.

In the solution of iRFP670 in the apoform (free of the chromophore) treated with chymotrypsin in the presence of ProteaseMAX™ Surfactant, short peptides with a size between 4.6 and 10 kDa were detected (Figure 4). According to the analysis of the amino acid sequence of iRFP670, only peptide A with a molecular mass of 5.04 kDa falls into this size range (Table S2). Complete hydrolysis by the protease of the rest of the apoprotein polypeptide chain is expected to yield peptides with a molecular mass of no more than 4.34 kDa (Table S2). In the case of incomplete hydrolysis of the apoprotein iRFP670, when cleavage sites Y213 and F247, or only F247, located at the intersecting sections of the polypeptide chain in the knot, are omitted, peptides with a molecular weight of 6.41 and 4.97 kDa (peptide B variants) should be formed, respectively (Table S2). This situation is likely realized in our case (Figure 4).

Our findings also highlight the possible function of the knot in the polypeptide chain of the chromophore-binding domain of phytochromes in increasing the resistance of these proteins to proteolytic degradation. Our data also show that the simultaneous covalent binding of BV to both domains of the NIR FPs with two cysteine residues Cys^PAS^ and Cys^GAF^ enhances significantly the stabilizing effect of the knot, making these proteins almost insensitive to the action of proteases.

## 3. Conclusions

In this work, we analyzed the effect of the covalent binding of BV to two cysteine residues in the PAS and GAF domains in NIR FPs on the structural and spectral properties of these proteins. Previously, the allosteric influence of protein monomers in dimeric NIR FPs on each other’s structure has been shown to govern the nature of BV binding in them [7]. Indeed, dimeric NIR FPs, in which only one of the cysteine residues in the PAS or GAF domains is present, have BV covalently bound to protein only in one monomer, while the chromophore in the second protein monomer is incorporated into the pocket of the GAF domain, but is not fixed by a covalent bond. The reason for this is that the covalent binding of BV in one monomer of the dimeric NIR FP bearing one cysteine residue may prevent the subsequent formation of a covalent bond between BV and another protein monomer. This explains the low molecular brightness of NIR FPs with one cysteine residue [7]. In dimeric NIR FPs with two cysteine residues, inhibition of covalent binding of the chromophore is not observed; BV in such proteins is covalently bound to both monomers. The absence of BV not covalently bound to the protein in NIR FPs with both cysteine residues Cys^PAS^ and Cys^GAF^ contributes to their higher molecular brightness compared to NIR FPs bearing one of these two cysteine residues or without them [7].

The analysis in the current work of several NIR FPs with two cysteine residues Cys^PAS^ and Cys^GAF^ corroborated with earlier studies of individual NIR FPs of this group [8,9], indicates that the quantum yield of their fluorescence is additionally affected by the covalent binding of BV simultaneously with two domains in these proteins, which leads to rigid fixation of the chromophore in their chromophore-binding pocket. It should be noted that, according to the literature, the kinetics of covalent binding of the chromophore depends on the number of cysteine residues in NIR FPs: the rate of covalent attachment of BV to Cys^GAF^ is significantly higher in proteins containing both Cys^GAF^ and Cys^PAS^ [38]. In that work, it has been suggested that as a result of rapid covalent fixation of BV in NIR FPs with two cysteine residues, they avoid the formation of non-fluorescent complexes with protoporphyrin in the cell, which increases their brightness [38]. Thus, several molecular mechanisms are responsible for the higher brightness of dimeric NIR FPs with two cysteine residues Cys^PAS^ and Cys^GAF^ compared to other BV-containing NIR FPs. 

Our data indicate that the covalent binding of BV simultaneously to two regions of the polypeptide chain, which additionally forms a figure-of-eight knot, in NIR FPs with two cysteine residues Cys^PAS^ and Cys^GAF^ leads to the screening of many sites of proteolytic degradation in them. As a result, covalent binding of BV in NIR FPs simultaneously with two cysteine residues Cys^PAS^ and Cys^GAF^ not only stabilizes their structure but also increases their resistance to proteolytic degradation in the cell, which is important for their use as fluorescent tags in the cell.

## 4. Materials and Methods 

### 4.1. Materials

Guanidine hydrochloride (GdnHCl), buffer components, and trypsin from bovine pancreas were purchased from Sigma (St. Louis, MO, USA) and used without further purification. Chymotrypsin and ProteaseMAX™ Surfactant (Trypsin Enhancer) were purchased from Promega (Madison, WI, USA).

### 4.2. Protein Expression and Purification

The target *NIR FP* genes, containing N-terminal polyhistidine tag, were amplified and cloned into a pBAD/His-B vector (Invitrogen, Waltham, MA, USA) using BglII and EcoRI sites expression in LMG194 host cells (Invitrogen, Waltham, MA, USA). NIR FPs are expressed and purified as previously described [7,39]. Briefly, NIR FPs in apo, (i.e., in the absence of a chromophore) and holoform, (i.e., in a complex with biliverdin) were obtained by expressing only the target protein gene and together with the hemoxygenase (HO) gene from the pWA23h-HO vector, respectively. The HO enzyme provides BV synthesis. The RM medium (48 mM Na_2_HPO_4_, 22 mM KH_2_PO_4_, 19 mM NH_4_Cl, 8.5 mM NaCl, 2% casamino acids, 1 mM MgCl_2_, 1 mM thiamine) supplemented with ampicillin was used for the expression of NIR FPs in the apoforms. Ampicillin and kanamycin were included in the culture medium for the expression of NIR FPs in holoform. Arabinose and rhamnose, respectively, were used for induction of the NIR FPs and HO expression. Cell lysates were purified with sequential affinity chromatography on a His Gravitrap column (GE Healthcare, Chicago, IL, USA) and ion-exchange chromatography on a MonoQ column (GE Healthcare, Chicago, IL, USA). The purity of the proteins was tested by SDS-PAGE using 15% polyacrylamide gels [40]. The protein solutions were dialyzed and stored in a 20 mM Tris/HCl solution, pH 8.0.

### 4.3. Spectral Measurements

Absorption spectra of NIR FPs were acquired using a U-3900H spectrophotometer (Hitachi, Tokyo, Japan) with microcells 101.016-QS 5 × 5 mm (Hellma, Müllheim, Germany).

The fluorescence spectra were measured using a Cary Eclipse spectrofluorimeter (Varian, Melbourne, Australia) with cells 10 × 10 × 4 mm (Starna, Atascadero, CA, USA). The raw fluorescence intensity of BV was corrected for the secondary inner filter effect [41]. The extinction coefficient of NIR FPs was determined by comparing the absorbance values at the far-red peak with the absorbance value at the Soret peak at about 390 nm. The extinction coefficient of tested proteins at the Soret band was supposed to be equal to that of free BV (39,900 M^−1^·cm^−1^). The fluorescence quantum yield of NIR FPs was calculated using iRFP713 as standard.

Tryptophan fluorescence spectra of NIR FPs were recorded using 295 nm excitation wavelengths and corrected for instrument sensitivity. The position of these spectra was determined by recording the parameter *A* = *I*_320_/*I*_365_, i.e., the ratio of fluorescence intensities on two slopes of the protein tryptophan fluorescence spectrum [42]. With this approach, a tiny spectral shift of 1 nm can be reliably detected.

Chromophore fluorescence decay curves NIR FPs were collected using a spectrometer FluoTime 300 (PicoQuant, Berlin, Germany) with the Laser Diode Head LDH-C-375 (λ_ex_ = 375 nm) or LDH-C-660 (λ_ex_ = 660 nm). The fitting of fluorescence decay curves was performed using the standard convolute-and-compare nonlinear least-squares procedure [43] with minimization according to Marquardt [44].

CD spectra were obtained using a Jasco-810 spectropolarimeter (Jasco, Tokyo, Japan). The far-UV CD spectra were recorded in a 1 mm path length cell from 250 to 190 nm, with a step size of 0.1 nm. The near-UV CD spectra were recorded in a 10 mm path length cell from 320 to 250 nm, with a step size of 0.1 nm. The visible CD spectra were scanned from 800 to 320 nm, with a step size of 0.1 nm, using a 10 mm path length cell. An average of three scans was obtained for all spectra. The CD spectra of the appropriate buffer solution were recorded and subtracted from the protein spectra.

### 4.4. Biochemical Measurements

The chromophore binding with NIR FPs was assayed by zinc-induced fluorescence and staining with Coomassie blue of protein samples separated by SDS-PAGE [45]. The content of NIR FPs with covalently attached BV was evaluated in ImageJ on the total intensity of the bands visualized with CB staining.

Protease-treated samples were analyzed by the 16/6% Tricine-SDS polyacrylamide gel electrophoresis, used to separate peptides over a wide range of molecular weights [28]. The peptides with a known molecular weight of up to 40 kDa were used as markers. Protein samples were incubated with a protease in a ratio of 20:1 overnight at 37 °C for trypsin and 50 °C for chymotrypsin (an optimum temperature for enzyme activity). Some samples were pre-incubated with urea at a concentration of 5 M at 50 °C for 30 min before adding the protease. Alternatively, samples were pre-incubated with 0.2, 0.1, and 0.025% ProteaseMAX™ Surfactant at 37 °C for 7 h without urea or with 2 M urea added. The samples thus prepared were mixed with proteases in a ratio of 20:1 and incubated overnight at 4, 20, 37, or 50 °C.

### 4.5. Analysis of Protein 3D Structure

The X-ray data of the PAS-GAF domains of the bacterial phytochrome *Rp*BphP2 (4E04.ent file [34] from PDB [46]) were used to analyze the location of proteolytic cleavage sites in NIR FPs.

## Figures and Tables

**Table 1:** Parameters of the chromophore fluorescence of NIR FPs in the holoform, containing Cys^PAS^ and Cys^GAF^, original and restored after GdnHCl-induced denaturation.

Parameter	iRFP713/V256C		iRFP670		BphP1-FP	
	Intact	Restored from 2.0 M GdnHCl	Intact	Restored from 2.5 M GdnHCl	Intact	Restored from 2.0 M GdnHCl
Absorbance maximum (nm)	662	662	644	646	643	643
Extinction coefficient at the main peak (M^−1^ cm^−1^)	94,000	98,500	110,000	111,000	66,800	69,400
Emission maximum (nm)	680	680–681	670	671	668	667
Quantum yield (%) ^1^	14.5	16.3	12.2	13.8	13.8	15.4
Chromophore time life (ns)	1.53	1.59	1.18	1.23	1.50	1.55
Tryptophan fluorescence maximum (nm)	333	330	329	328	330	329

^1^ The value of fluorescence quantum yield of original NIR FPs is taken from [7,12].

**Table 2:** Estimated size of BV-containing peptides obtained by partial and complete cleavage of iRFP670 in the holoform with chymotrypsin (cleavage after residues F, Y, and W).

Peptides with BV, kDa		Peptide B Sequence at Complete Cleavage or When Some Cleavage Sites are Skipped by the Protease
**A-BV-B ^1^**	**BV-B**	
**partial cleavage ^3^**		
12.04	6.99	PASLVPQQARLL**Y_213_**LKNAIRVVSDSRGISSRIVPEHDASGAALDLS**F_247_**AHRSISPCys^GAF^HLE **F_260_** ^2^
14.28	9.24	PASLVPQQARLLY_213_LKNAIRVVSDSRGISSRIVPEHDASGAALDLS**F_247_**AHRSISPCys^GAF^HLE **F_260_**LRNMGVSASMSLSIIIDGTLW_281_
15.22	10.18	PASLVPQQARLL**Y_213_**LKNAIRVVSDSRGISSRIVPEHDASGAALDLS**F_247_**AHRSISPCys^GAF^HLE **F_260_**LRNMGVSASMSLSIIIDGTL**W_281_**GLIICHHY_289_
**complete cleavage**		
7.13	2.09	AHLRSISPCys^GAF^HLEF_260_

^1^ Peptide A contains Cys^PAS^ (its size is 5.04 kDa, sequence—MARKVDLTSCys^PAS^DREPIHIPGSIQPCGCLLACDAQAVRITRITENAGAF_52_); peptide B contains Cys^GAF^. ^2^ numbering corresponds to sequence alignment of iRFP670 relative to *Rp*BphP2 (4E04 file in PDB [34]). ^3^ cleavage sites skipped by the protease are in bold.

## Data Availability

The data that support the findings of this study are available from the corresponding author upon reasonable request.

## Supplementary Materials

Supplementary materials can be found at https://www.mdpi.com/article/10.3390/ijms23137347/s1.
