# Supplementary material for: Impact of Double Covalent Binding of BV in NIR FPs on Their Spectral and Physicochemical Properties
Source: Int J Mol Sci. 2022 Jul 1;23(13):7347. doi: 10.3390/ijms23137347 (PMC9267011; doi:10.3390/ijms23137347)
Supplement: Supplementary file 1 [file ijms-23-07347-s001.zip › ijms-1785925-supplementary.pdf]

Supplementary Information

Impact of double covalent binding of BV in NIR FPs on their spectral and physicochemical properties

Olga V. Stepanenko,[a] Irina M. Kuznetsova,[a] Konstantin K.Turoverov [a,*] and Olesya V. Stepanenko[a]

[a]Laboratory of Structural Dynamics, Stability and Folding of Proteins, Institute of Cytology, Russian Academy of Sciences, 4 Tikhoretsky Avenue, 194064 St. Petersburg, Russia

*Corresponding authors

Table S1. Molecular weight of peptides obtained by complete cleavage of iRFP670 in the holoform with chymotrypsin (cleavage after residues F, Y, and W) and trypsin (cleavage after residues K and R).

	**peptides, kDa**
**chymotrypsin**	7.13[1, *] (*peptide A-BV-peptide B*); 5.04[2] (*peptide A*); 4.34; 3.48; 2.69; 2.26; 2.09[3, *] (*BV-peptide B*); 2.08; 1.80; 1.74; 1.58; 1.46; 1.12; 1.02; 0.96; 0.82; 0.79; 0.62; 0.6; 0.35; 0.32; 0.26; 0.17
**trypsin**	3.31; 2.79 (*peptide A-BV-peptide B*); 2.49; 2.40; 2.38; 2.33; 2.12; 1.99; 1.88 (*BV-peptide B*); 1.40; 1.25; 1.18; 1.03; 0.99; 0.96; 0.91 (*peptide A*); 0.91; 0.77; 0.68; 0.66; 0.65; 0.62; 0.60; 0.52; 0.50; 0.47; 0.39; 0.38; 0.37; 0.25; 0.17; 0.15

[1] BV is covalently bound to two cysteines: Cys^{PAS} of peptide A and Cys^{GAF} of peptide B;
[2] peptide A contains Cys^{PAS};
[3] peptide B contains Cys^{GAF} to which BV is covalently attached;
[*] the corresponding peptides contain a covalently attached BV and can be identified by Zn-induced fluorescence

Table S2. Molecular weight of peptides obtained by cleavage of iRFP670 in the apoform with chymotrypsin (cleavage after residues F, Y, and W).

	**complete cleavage**	**partial cleavage**
**peptides, kDa**	5.04 [1] (*peptide A*); 4.34; 3.48; 2.69; 2.26; 2.08; 1.80; 1.74; 1.58; 1.51[2] (*peptide B*); 1.46; 1.12; 1.02; 0.96; 0.82; 0.79; 0.62; 0.6; 0.35; 0.32; 0.26; 0.17	6.41 or 4.97[3]

[1] peptide A contains Cys^{PAS};
[2] peptide B contains Cys^{GAF};
[3] peptide B, formed if the polypeptide chain is not cleaved at sites Y213, F247. Numbering corresponds to sequence alignment of iRFP670 relative to *Rp*BphP2 (4E04 file in PDB [1]).

```
              1        10        20        30        40        50        60
RpBphP2(4E04) MTEGSVARQP DLSTCDDEPI HIPGAIQPHG LLLALAADMT IV-AGSDNLP ELTGLAIGAL
iRFP670       MARK-----V DLTSCDREPI HIPGSIQPCG CLLACDAQAV RITRITENAG AFFGRETPRV
              ---------- ---------- ---------- ---------- ---------- ----------
                                     PAS

                      70        80        90       100       110       120
RpBphP2(4E04) IGRSAADVFD SETHNRLTIA LAEPGAAVGA PIAVGFTMPD GERAFNGSWH RHDQLVFLEL
iRFP670       GELLADYFGE TEAHALRNAL AQSSDPKRPA LIFGWRDGLT GRTFD-ISLH RHDGTSIIEF
              ---------- ---------- ---------- ---------- ---------- ----------
                                     PAS

                     130       140       150       160       170       180
RpBphP2(4E04) EPPQRDVRYP QAFFRSVRSA IRRLQAAETL ESACAAAAQE VREITGFDRV MIYRFASDFS
iRFP670       EPAAAEQADN PL--RLTRQI IARTKELKSL EEMAARVPRY LQAMLGYHRV MLYRFADDGS
              -------___ __________ __________ __________ __________ __________
              PAS     GAF

                     190       200       210       220       230       240
RpBphP2(4E04) GEVIAEDRCA EVESYLGLHF PASDIPAQAR RLYTINPVRI IPDINYRPVP VTPDLNPRTG
iRFP670       GMVIGEAKRS DLESFLGQHF PASLVPQQAR LLYLKNAIRV VSDSRGISSR IVPEHDAS-G
              __________ __________ __________ __________ __________ __________
                                     GAF

                     250       260       270       280       290       300
RpBphP2(4E04) RPIDLSFAIL RSVSPVHLEY MRNIGMHGTM SISILRGERL WGLIACHHRK PNYVDLEVRQ
iRFP670       AALDLSFAHL RSISPCHLEF LRNMGVSASM SLSIIIDGTL WGLIICHHYE PRAVPMAQRV
              __________ __________ __________ __________ __________ __________
                                     GAF

                     310       320
RpBphP2(4E04) ACELVAQVLA WQIGVMEEQA
iRFP670       AAEMFADFLS LHFTAAHHQR
              __________ __________
                         GAF
```

Figure S1. Alignment of amino acid sequences of iRFP670 with their wild-type templates *Rp*BphP2-PAS-GAF. The highlighted in magenta are the conservative Cys residue in the PAS domain (Cys15) and the unique Cys residue in the GAF domain of iRFP670 (Cys256). The amino acids Phe, Tyr and Trp, preceding the chymotrypsin-cleavable amide bond, and Lys and Arg, preceding the trypsin-cleavable amide bond, are highlighted in red and green, correspondingly.

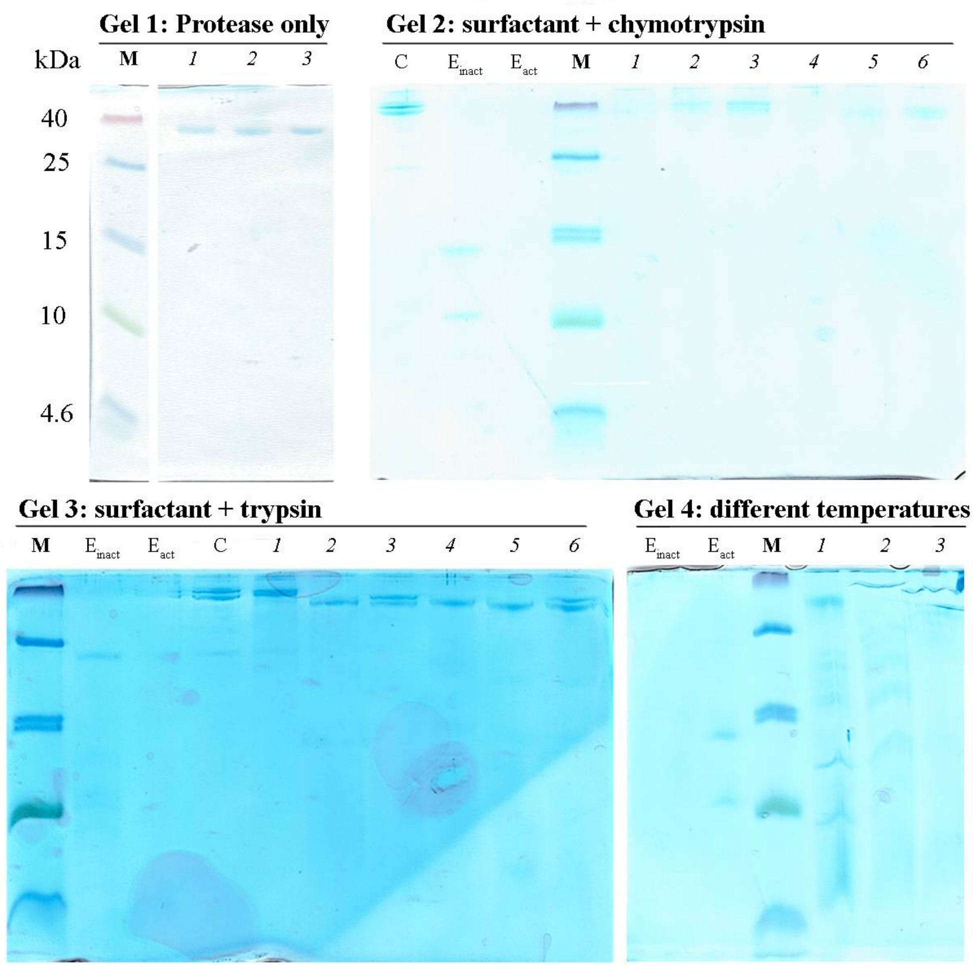

Figure S2. Cleavage of iRFP670 in the holoform under different conditions.
Tricine-SDS PAGE of protein samples followed by staining with Coomassie blue (CB). The following conditions were checked:

Gel 1, lanes 1 and 2 – protein cleavage with chymotrypsin at 50 °C without and with preliminary incubation of samples in the presence of 5 M urea; lane 3 – protein cleavage by trypsin at 37 °C.

Gel 2, lanes 1, 2, and 3 – protein cleavage with chymotrypsin at 50 °C with preliminary incubation of samples in the presence of 2 M urea and ProteaseMAX™ Surfactant at concentrations of 0.2, 0.1, and 0.025%; lanes 4, 5, and 6 – protein cleavage with chymotrypsin at 50 °C with preliminary incubation of samples in the presence of ProteaseMAX™ Surfactant at concentrations of 0.2, 0.1, and 0.025%.

Gel 3, lanes 1, 2, and 3 – protein cleavage with trypsin at 37 °C with preliminary incubation of samples in the presence of 2 M urea and ProteaseMAX™ Surfactant at concentrations of 0.2, 0.1, and 0.025%; lanes 4, 5, and 6 – protein cleavage with trypsin at 37 °C with preliminary incubation of samples in the presence of ProteaseMAX™ Surfactant at concentrations of 0.2, 0.1, and 0.025%.

Gel 4, lanes 1, 2, and 3 – protein cleavage with chymotrypsin at 4, 37, and 50 °C with preliminary incubation of samples in the presence of 0.2% surfactant.

M – marker peptides, C – control samples of the original protein. The activity of proteases is confirmed by their self-cleavage at the optimum temperature. (E_{act}). Solutions of proteases whose activity is blocked (by addition of a PMSF inhibitor at a concentration of 1 mM at 4 °C) contain only peptides with a molecular weight of about 14 and 10 kDa for chymotrypsin and 23.3 kDa for trypsin, which corresponds to intact emzyme subunits (E_{inact}).

References

[1] D. Bellini, M.Z. Papiz, Acta Crystallogr D Biol Crystallogr, Dimerization properties of the RpBphP2 chromophore-binding domain crystallized by homologue-directed mutagenesis, 68 (2012) 1058-1066.
